# Genetic and Clinical Insights into ALS/FTD: Profiling a Rare Cohort to Explore Spectrum Heterogeneity

**DOI:** 10.3390/jpm15100451

**Published:** 2025-09-28

**Authors:** Ana Marjanovic, Elka Stefanova, Vanja Viric, Aleksa Palibrk, Gorana Mandić Stojmenović, Tanja Stojković, Lenka Stojadinovic, Ivana Basta, Ivana Novakovic, Zorica Stević, Milena Jankovic

**Affiliations:** 1 Neurology Clinic, University Clinical Centre of Serbia, 11000 Belgrade, Serbia; ana.marjanovic@yahoo.com (A.M.); steela21@gmail.com (E.S.); vanja.viric97@gmail.com (V.V.); palibrk17@gmail.com (A.P.); goranamandic@yahoo.com (G.M.S.); tanjili80@gmail.com (T.S.); ivanabasta@yahoo.com (I.B.); zstevic05@gmail.com (Z.S.); 2 Faculty of Medicine, University of Belgrade, 11000 Belgrade, Serbia; lenkastojadinovic@gmail.com (L.S.); tetaana61@yahoo.com (I.N.)

**Keywords:** ALS/FTD, *C9orf72*, *ATXN1*, *ATXN2*

## Abstract

**Background**: Amyotrophic lateral sclerosis (ALS) and frontotemporal dementia (FTD) are recognized as a spectrum of neurodegenerative disorders with overlapping clinical, pathological, and genetic features. The identification of *C9orf72* hexanucleotide repeat expansion as the most common genetic cause of both conditions has prompted further investigation of genetic modifiers that may contribute to disease heterogeneity. We aimed to analyze the frequency of *C9orf72* repeat expansions and potential modifying roles of *APOE*, *ATXN1*, and *ATXN2* in Serbian ALS/FTD patients. **Methods**: Our study included an ALS/FTD cohort (*n* = 22) and healthy controls (n = 94). Repeat sizing in *C9orf72, ATXN1* and *ATXN2* was performed by fluorescent polymerase chain reaction (PCR) and capillary electrophoresis, while repeat-primed PCR was used to confirm *C9orf72* expansions. *APOE* genotyping was conducted using real-time PCR assays targeting SNPs rs429358 and rs7412. **Results**: In the ALS/FTD cohort, 31.82% of the patients had heterozygous *C9orf72* repeat expansion. The most common *APOE* genotype among patients was ε3/ε3 (72.73%). Intermediate-length *ATXN1* alleles (32–44 repeats) were detected in 13.64% of patients and *ATXN2* intermediate-length alleles (27–33 repeats) were found in 9% of patients. No significant differences were observed between ALS/FTD patients and controls in *APOE ε4* frequency or intermediate *ATXN1/ATXN2* repeats. **Conclusions**: Larger, population-specific studies and meta-analyses are needed to better understand the role of genetic modifiers in ALS/FTD pathogenesis and their influence on clinical heterogeneity. By integrating genetic and clinical data, this study represents a step toward the development of precision medicine strategies for ALS/FTD.

## 1. Introduction

Amyotrophic lateral sclerosis (ALS) is a progressive neurodegenerative disorder primarily affecting motor neurons, leading to muscle weakness, general paralysis, and death, typically within 3–5 years after symptom onset [1]. Frontotemporal dementia (FTD) is the second most common form of early-onset dementia, characterized by changes in language, behavior, and executive function [2,3]. ALS and FTD are nowadays considered to represent two ends of a shared disease spectrum, with overlapping clinical, pathological, and genetic features, commonly referred to as the ALS–FTD spectrum [4]. In clinical presentation, in addition to motor symptoms, approximately 50% of ALS patients exhibit signs of cognitive impairment, and about 20% fulfill the diagnostic criteria for FTD [5]. Also, co-morbid Alzheimer’s dementia (AD) is described in ~ 2% of ALS patients [6]. When observing patients with FTD, 14% have a clinical diagnosis of definite ALS, and 36% have some ALS clinical features [7]. ALS/FTD is not a separate diagnostic entity in current nosology, but rather a co-occurrence of ALS and FTD, based on established criteria for each. ALS/FTD is diagnosed when patients meet El Escorial criteria for ALS and Rascovsky (bvFTD) or Gorno-Tempini (PPA) criteria for FTD simultaneously [8,9,10]. According to the Strong et al. consensus from 2017, ALS/FTD represents the most severe form of the ALS–FTD spectrum, characterized by the coexistence of motor neuron disease and frontotemporal dementia [11].

ALS and FTD are both individually rare disorders—ALS occurs in roughly 2.08 per 100,000 person-years and FTD in about 2.4 per 100,000 person-years [12,13]. Consequently, the overlap phenotype ALS/FTD is relatively infrequently present within the already-rare disease spectrum.

The *C9orf72* gene’s repeat expansions have been established as the most common genetic cause of both ALS and FTD [14,15] and are identified in around 30% of individuals presenting with the combined ALS/FTD phenotype [16]. The clinical presentation associated with *C9orf72* repeat expansions is highly variable and remains difficult to predict. In addition, *C9orf72* repeat expansions have also been reported in other neurodegenerative and psychiatric disorders, including Parkinson’s disease, Alzheimer’s disease, and movement disorders, albeit at lower frequencies (Table 1) [16].

In recent years, increasing attention has been directed toward identifying genetic modifiers that influence the clinical phenotype of *C9orf72*-associated disease; however, findings across studies have been inconsistent and often conflicting [24,25,26,27,28,29]. APOE ε4 genotype is considered a risk factor in late-onset Alzheimer’s disease in both familial and sporadic cases [30]. In contrast, APOE ε2 has been suggested to increase the risk of frontotemporal dementia in patients with ALS, whereas the ε4 allele does not appear to confer a similar effect [24]. Intermediate-length polyglutamine repeats in both *ATXN1* and *ATXN2*-genes associated with spinocerebellar ataxias (SCAs) have also been observed in ALS, in both *C9orf72* repeat expansion carriers and non-carriers [25,26].

Despite these important findings, the current literature on genetic modifiers in ALS/FTD remains relatively sparse, with most studies focusing on ALS or FTD patient groups. Consequently, the role of genetic modifiers specifically in patients presenting with the combined ALS/FTD phenotype has not been comprehensively explored. To address this gap, our study focused exclusively on individuals diagnosed with ALS/FTD, thereby providing a more homogeneous cohort for evaluating the potential modifying effects of APOE genotype and intermediate polyglutamine repeats in *ATXN1* and *ATXN2*.

This study investigates clinical and demographic characteristics, *C9orf72* expansion status, APOE genotypes, and *ATXN1/ATXN2* repeat lengths in ALS/FTD patients and controls, with a comparative analysis between carriers and non-carriers of the *C9orf7*2 repeat expansion.

## 2. Material and Methods

The study included 22 patients diagnosed with ALS/FTD according to the revised El Escorial criteria for ALS [8] and the Rascovsky criteria for behavioral variant FTD [10] or the Gorno-Tempini criteria for primary progressive aphasia [9]. Patients fulfilling both sets of criteria were classified as ALS/FTD, consistent with the consensus framework [11]. The patients were recruited at the Neurology Clinic, University Clinical Centre of Serbia (UCCS). In addition, 94 healthy individuals were included as controls. All participants provided written consent after being informed about the genetic testing, and ethical approval was obtained from the UCCS Ethics Committee.

Genomic DNA was extracted from peripheral blood samples using standard protocols. Analysis of the *C9orf72* hexanucleotide (GGGGCC) repeat and trinucleotide CAG repeat sizing in *ATXN1* and *ATXN2* was performed using fluorescent polymerase chain reaction (PCR) followed by capillary electrophoresis on an ABI 3500 DNA Genetic Analyzer (Applied Biosystems, Waltham, MA, USA). For all homozygous *C9orf72* alleles, repeat-primed PCR was additionally performed. Primer sequences for *C9orf72* and *ATXN1* were designed in-house using Primer3 software version 3.0 [31], while previously published primers and protocols were applied for *ATXN2* and *C9orf72* repeat-primed PCR [14,15]. The primer sequences are listed in Table 2.

A threshold of ≥30 repeats was used to define a pathogenic *C9orf72* expansion [14], and all the expansion carriers were confirmed using Southern blot analysis [33]. The threshold for pathogenic *ATXN1* expansion is defined as ≥44 repeats and for *ATXN2* >33 repeats, while intermediate repeats with uncertain clinical significance are considered 32–43 for *ATXN1* [34] and 27–33 for *ATXN2* [35].

*APOE* genotyping was performed by real-time PCR with TaqMan assays targeting SNPs rs429358 and rs7412 (Thermo Scientific, Waltham, MA, USA).

For all patients, data were collected on age at symptom onset, gender, family history (including ALS, dementia, Parkinson’s disease, and psychiatric disorders), and the site of motor symptom onset.

Statistical analysis was performed using the Statistica program (v.12). Analysis of variance was used to compare continuous variables, while categorical variables among patients and the control group, as well as between patients with and without *C9orf72* repeat expansion, were analyzed using the Chi-squared test. A *p*-value of < 0.05 was considered significant.

## 3. Results

### 3.1. ALS/FTD Cohort

This study included 22 patients with ALS/FTD overlap phenotype (54.55% male). A positive family history was registered in 27.27% of patients and a negative one in 45.45%, and for 27.27% of patients, the data about the family history were not available.

The average age of onset of our cohort was 59.59 ± 8.89 (95%CI: 55.65–63.53), and the range 42.0–74.0 years. When we compared the age of onset in our group, no notable differences were observed between genders or between patients with spinal vs. bulbar onset. Bulbar onset was registered in 50% of patients, while the bvFTD was the most common clinical presentation in the analyzed cohort.

**Figure 1:**
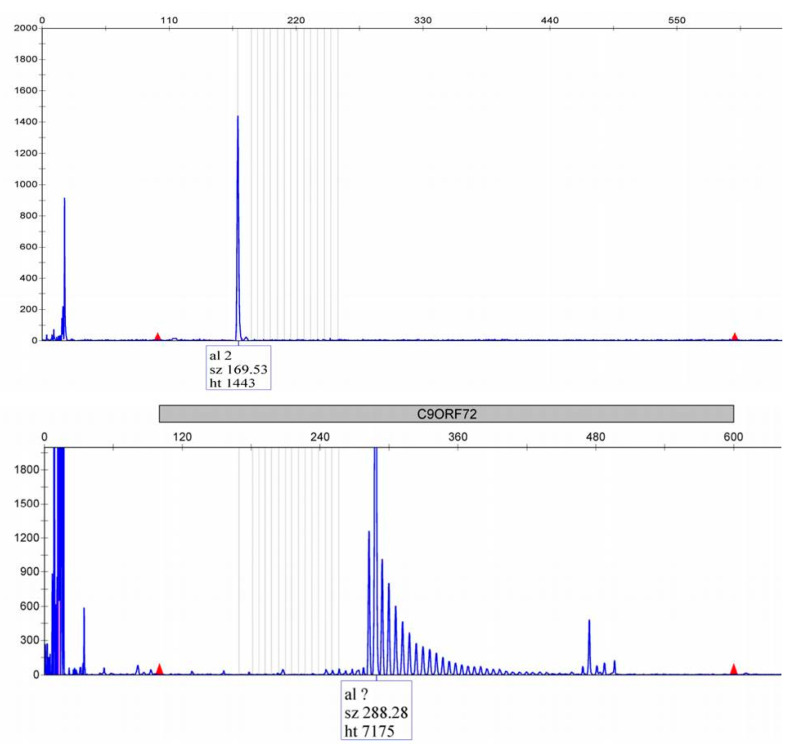
Electropherogram of the *C9orf72* fluorescent sizing PCR in ALS/FTD patient: upper electropherogram—patient carrying normal allele (2 repeats); bottom electropherogram—repeat-primed PCR detected *C9orf72* repeat expansion. “?” — the bins were not set for the expanded alleles. Red: red markers indicate the range for the fluorescent signal detection.

**Figure 2:**
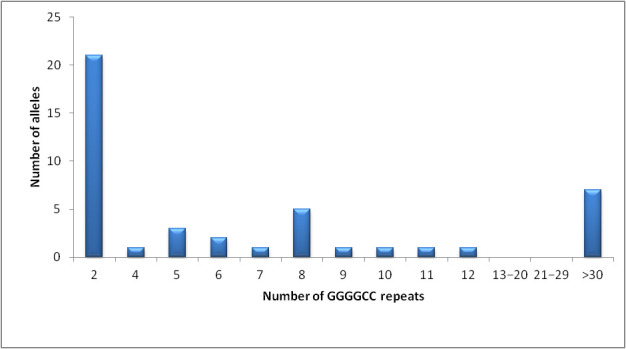
The distribution of *C9orf72* allele sizes in ALS/FTD patients.

**Figure 3:**
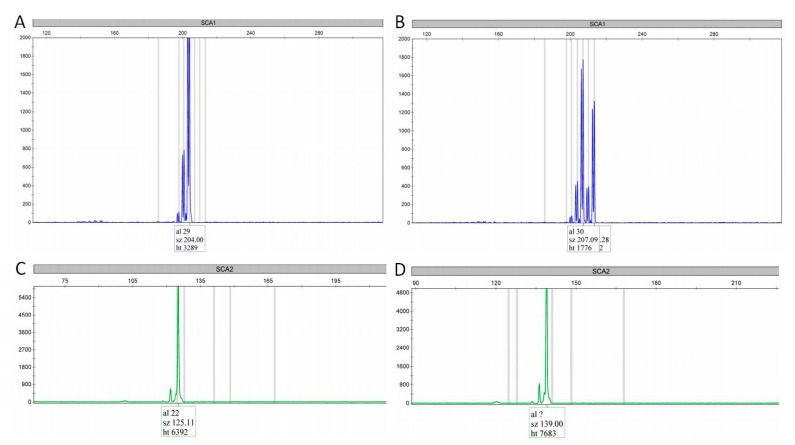
Electropherograms of *ATXN1* and *ATXN2* repeats. (**A**) patient with 29 *ATXN1* normal repeats (homozygous); (**B**) patient with 30/32 *ATXN1* intermediate repeats; (**C**) patient with 22 *ATXN2* normal repeats (homozygous); (**D**) patient with 27 *ATXN2* intermediate repeats (homozygous; “?” — bin for the allele with 27 repeats was not set as the repeat count could be assessed according to the allele size).

**Figure 4:**
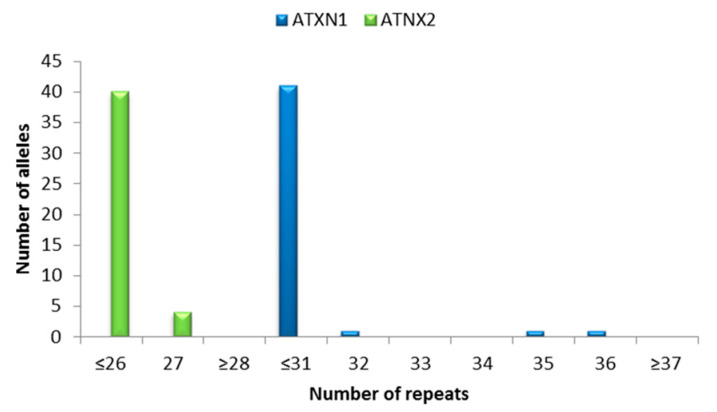
*ATXN1* and *ATXN2* allele distribution in ALS/FTD patients.

In this study, we registered 31.82% of patients with heterozygous *C9orf72* repeat expansion (57.14% males). Examples of normal and expanded *C9orf72* repeats are shown in Figure 1, while the distribution of *C9orf72* repeats is presented in Figure 2. No pathogenic repeat expansions were observed in *ATXN1* or *ATXN2*, as anticipated. Instead, our analysis focused on intermediate-length alleles, which have been implicated as modifiers of ALS/FTD phenotypes (Figure 3). *ATXN1* intermediate repeats (32–44 CAG) were present in 13.64% of patients, while *ATXN2* (27–33 CAG) repeats were present in 9% of the patients (Figure 4). One patient carried intermediate repeats in both *ATXN1* and *ATXN2*. The disease presented with spinal-onset ALS accompanied by bvFTD at the age of 72. The patient also had a positive family history of dementia, but no additional specific clinical features were observed.

**Figure 5:**
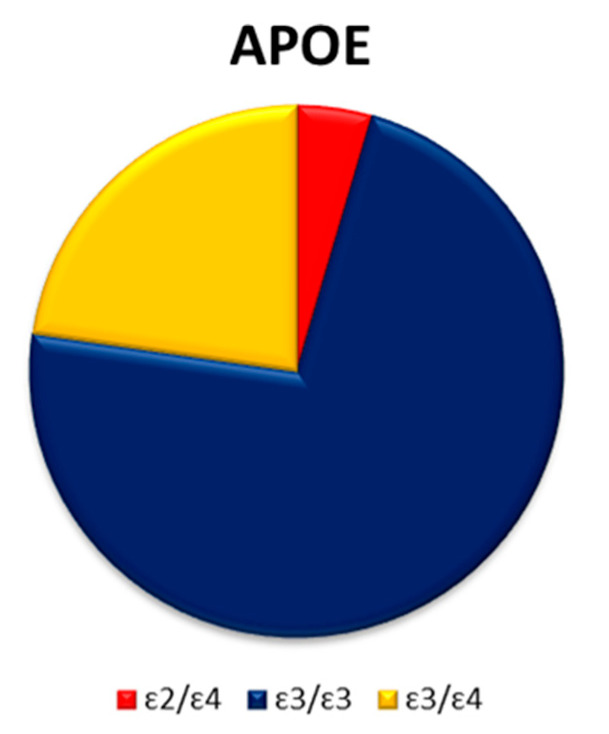
APOE genotype distribution in ALS/FTD patients.

The most common *APOE* genotype in the cohort was ε3/ε3, present in 72.73% followed by ε3/ε4 in 22.73% of the patients. Genotype ε2/ε4 was registered in only one patient (Figure 5).

### 3.2. ALS/FTD Cohort vs. Control Group

The presence of the *C9orf72* repeat expansion was not registered in the control group. *ATXN1* intermediate repeats were present in 30.85% (13.79% in homozygous form), while *ATXN2* in 8.51% (37.5% in homozygous form). Among controls, *APOE ε3/ε3* was the most common present in 74.47%, while *ε4/ε4* was registered in 2.13%. Statistical analysis showed no significant difference between patients and controls in the frequency of *APOE ε4*, intermediate repeats in *ATXN1* and/or *ATXN2* (*p* > 0.05).

### 3.3. ALS/FTD C9orf72 Expansion Positive vs. C9orf72 Negative Patients

Among *C9orf72* repeat expansion carriers positive and negative family histories were each documented in an equal number of patients (42.86%), while family history information was not available for one individual.

The average age of onset of the expansion carriers was 56.43 ± 9.00 (95% CI: 48.11–64.75) with a range of 42–67 years. Among the expansion carriers, there were no significant differences in age of onset based on gender or type of disease onset (spinal vs. bulbar). Regarding the site of onset, 3 patients each had spinal or bulbar onset. For one patient, data about the site of onset were not available (Table 3).

When we compared patients with and without the expansion, there was no significant difference in gender and disease onset frequencies, as well as the presence of positive family history, nor between the average ages of onset (*p* ˃ 0.05) (Table 4).

In *C9orf72* positive patients, the most common genotype was ε3/ε3 registered in 57.14% followed by ε3/ε4 in 42.86% of the patients. Among patients without the expansion 80% had ε3/ε3, 13.33% had ε3/ε4, and only one patient had ε2/ε4. Statistical analysis did not show any significant difference in the presence of ε4 allele in *C9orf72* positive and negative patients (*p* = 0.262). Intermediate-length repeats in the *ATXN1* gene were identified in only one *C9orf72* expansion carrier, whereas no intermediate repeats were observed in the *ATXN2* gene. The patient who had *C9orf72* repeat expansion and intermediate *ATXN1* repeat number had disease onset at the age of 62 with bulbar symptoms and positive family history for ALS.

## 4. Discussion

In this study, we characterized the clinical and demographic features, frequency of the *C9orf72* repeat expansion, APOE genotype, *ATXN1,* and *ATXN2* repeat size in ALS/FTD patients from Serbia.

Our analyzed cohort included 22 patients, and the observed *C9orf72* repeat expansion prevalence of 31.82% among ALS/FTD cases aligns with previous reports in other populations [36,37], as well as with review data indicating a frequency of approximately 30% [16].

Within our cohort, the highest rate of expansion carriers (50%) has been found among patients with positive family history, as previously reported in other studies [38,39]. Interestingly, 30% of expansion carriers were identified among patients with a negative family history. Although incomplete data and unavailable medical records for some relatives must be considered, this rate is considerably higher than the previously reported 6% in a similarly sized cohort [37]. This high rate of the expansion carriers in our patients with negative family history emphasizes the significance of the genetic testing in ALS/FTD cases, even in the absence of relatives with ALS and/or dementia in the family.

The average age of onset among *C9orf72* expansion carriers in our cohort was 56.43 years, with no significant difference compared to patients without the expansion. Byrne et al. have analyzed the similarly sized group (n = 30), and reported a younger age at symptom onset among expansion carriers [36]. Additionally, the distribution of genders in our cohort did not differ between expansion carriers and non-carriers, consistent with findings from the Byrne et al. study [36].

No significant differences in the frequency of the *APOE* ε4 allele were observed between patients and controls. In this study, the *APOE ε3/ε3* genotype was the most prevalent in both expansion carriers, non-carriers, and in the entire cohort. These findings are in concordance with previous reports [24,40]. Data on *APOE* genotypes in ALS/FTD patients with *C9orf72* expansion are limited. However, studies that examined *APOE* in *C9orf72* expansion carriers, whether diagnosed with ALS or FTD, have similarly reported ε3/ε3 as the most common genotype [41,42]. While APOE ε4 is recognized as a genetic risk factor for Alzheimer’s disease (AD) [30], its frequency among *C9orf72* expansion carriers with clinically and pathologically confirmed AD was almost equally represented in relation to other alleles [43]. The clinical significance of *APOE* in ALS and FTD remains inconclusive, with conflicting findings reported in the literature [41,44,45,46,47].

In our ALS/FTD cohort, the frequencies of intermediate *ATXN1* and *ATXN2* repeats did not differ from those observed in the control group. Only one patient carried both the *C9orf72* expansion and an intermediate *ATXN1* repeat. Expansion of the CAG repeats above 44 in the *ATXN1* gene causes spinocerebellar ataxia type 1 (SCA1), while more than 35 CAG repeats in the *ATXN2* gene is the cause of spinocerebellar ataxia type 2 (SCA2) [48]. Intermediate length CAG repeats in both genes have been reported in association with ALS [25,35]. *ATXN1* intermediate repeats have a strong association with ALS carrying the *C9orf72* repeat expansion, being present in 15.82–19.6% of these individuals [25,49]. Among familial *C9orf72* cases, the frequency is nearly twice as high compared to sporadic cases (27.8% FALS vs. 15.15% SALS), although not significantly different. The link between *ATXN1* and *C9orf72* was proposed towards developing ALS in *C9orf72* positive patients [49]. The study in a French cohort (n = 168) with ALS/FTD showed a significant association of ≥29 CAG repeats in *ATXN2* with ALS/FTD, especially with the familial form, demonstrating the risk of developing ALS/FTD. Intermediate *ATXN2* repeats were found in *C9orf72* expansion carriers both in ALS and ALS/FTD patients, but absent in FTD, suggesting that *ATXN2* CAG expansion might act as a disease modifier towards ALS of the ALS/FTD spectrum [26]. Italian FTD study (n = 368) confirmed that intermediate repeats in *ATXN2* are not associated with FTD but may modify the clinical features of the disease, e.g., younger age of onset, and increased presence of parkinsonism and psychotic symptoms at disease onset [50]. These literature findings suggest that polygenic background, epigenetic regulation, and environmental factors (such as toxins, lifestyle, and inflammation) may influence whether a patient develops motor neuron disease or cerebellar ataxia.

## 5. Conclusions

The primary limitation of our study is the modest sample size, which consequently restricts our ability to make generalizable conclusions about the influence of genetic variants on phenotypic expression. However, due to the rarity of *C9orf72* repeat expansion-associated ALS/FTD, publishing data from small cohorts remains important to gradually build knowledge in this field. Considering the complexity of clinical manifestations in *C9orf72* repeat expansion carriers, further studies with larger cohorts are necessary to elucidate the potential contribution of APOE genotype, as well as *ATXN1* and *ATXN2* repeat lengths, to the ALS/FTD phenotype.

Nonetheless, our findings emphasize the necessity of *C9orf72* genetic testing for all patients exhibiting the ALS/FTD phenotype, irrespective of family history.

## Figures and Tables

**Table 1:** Clinical presentations associated with *C9orf72* hexanucleotide repeat expansions.

Clinical Domain	Main Clinical Features with HP Terms [17]	Comments/Distinctions	References
ALS	Limb- or bulbar-onset weakness (HP:0003690; HP:0001283), UMN and LMN signs (HP:0002127; HP:0002366), early respiratory involvement (HP:0002098)	Most common phenotype; often positive family history	[14,15]
FTD	Executive dysfunction (HP:0033051), frontal memory disorder (HP:0100543), language impairment (HP:0002463), changes in behavior (HP:0000708) aphasia (HP:0002381), apathy (HP:0000741), disinhibition (HP:0000734), loss of empathy (HP:5200037), compulsive behaviors (HP:0000722)	bvFTD is the most frequent dementia subtype non-fluent/agrammatic PPA is less common than bvFTD	[18,19,20]
Overlap phenotypes	ALS/FTD—concomitant motor and cognitive/behavioral impairment (HP:0100543; HP:0000708) FTD/PSP—behavioral an cognitive changes (HP:0100543; HP:0000708) accompanied with motor and oculomotor deficits (HP:000605)	ALS/FTD reported in up to ~40–50% of carriers	[14,15,18,21]
Psychiatric features	Delusions (HP:0000746), psychosis (HP:0000709), hallucinations(HP:0000738), mood disorders (HP:0000712), paranoid schizophrenia (HP:0100753)	May precede neurological signs by years	[18,19]
Movement disorders	Parkinsonism (HP:0001300), motor stereotypes (HP:0000733), chorea (HP:0002072), oromandibular dyskinesia (HP:0012048), ataxia (HP:0001251), dystonia (HP:0001332), myoclonus (HP:0001336), tremor (HP:0001337)	Occasionally isolated or combined with ALS and/or FTD	[18,22,23]
Other dementias	Alzheimer’s disease, Unspecified dementia, Corticobasal degeneration, Lewy body dementia, sporadic Creutzfeld-Jacobs disease	Reported at a very low frequency	[16,18]

HP—The Human Phenotype Ontology (HPO) data base terms; UMN—upper motor neuron; LMN—lower motor neuron; bvFTD—behavioral variant of FTD; PPA—primary progressive aphasia; PSP—progressive supranuclear palsy.

**Table 2:** The primer sequences for fluorescent PCR.

	Primer Sequences	Reference
*C9orf72*	C9F: FAM-GAAACAACCGCAGCCTGTAG C9R: GCCTCCTCACTCACCCACT	in-house
*C9orf72* repeat-primed PCR	C9orf72F: FAM-AGTCGCTAGAGGCGAAAGC C9orf72R: TACGCATCCCAGTTTGAGACGGGGGCCGGGGCCGGGGCCGGGG C9orf72A: TACGCATCCCAGTTTGAGACG	[14]
	MRX-F: NED-TGTAAAACGACGGCCAGTCAAGGAGGGAAACAACCGCAGCC MRX-M13R: CAGGAAACAGCTATGACC MRX-R1:CAGGAAACAGCTATGACCGGGCCCGCCCCGACCACGCCCCGGCCCCGGCCCCGG	[15]
*ATXN1*	SCA1F: CCAACATGGGCAGTCTGAG SCA1R: FAM-TGGACGTACTGGTTCTGCTG	in-house
*ATXN2*	SCA2F: GGGCCCCTCACCATGTCG SCA2R: VIC-CGGGCTTGCGGACATTGG	[32]

F—forward primer; R—reverse primer; A—anchor primer; M13—standard DNA sequence used as primer; FAM, NED and VIC—fluorescent dyes.

**Table 3:** Clinical and Genetic Features of ALS/FTD Cohort.

Characteristic	ALS/FTD (n = 22)	C9orf72+ (n = 6)	C9orf72– (n = 15)
Demographics and Genetic Features			
Gender (male %)	90%	100%	85.7%
GGGGCC expansion	3 (30%)	–	–
Clinical Features			
Limb weakness (HP:0003690)	10 (45.5%)	3 (42.9%)	7 (46.7%)
Upper extremity (UE, HP:0003484)	9 (90%)	2 (66.7%)	7 (100%)
Left	1 (11.1%)	1 (50%)	–
Right	3 (33.3%)	1 (50%)	2 (28.6%)
Both	5 (55.6%)	–	5 (71.4%)
Lower extremity (LE, HP:0007340)	1 (10%)	1 (33.3%)	–
Left	1 (100%)	1 (100%)	–
Bulbar weakness (HP:0001283)	11 (50%)	3 (42.9%)	8 (53.3%)

HP—The Human Phenotype Ontology (HPO) data base terms [17].

**Table 4:** Comparison of clinical-demographic characteristics of ALS/FTD patients with and without *C9orf72* repeat expansion.

	*C9orf72* Expansion Positive	*C9orf72* Expansion Negative	*p* Value
Gender			0.867
male	57.14%	53.33%	
female	42.86%	46.67%	
Onset			0.890
spinal	50%	46.67%	
bulbar	50%	53.33%	
Positive family history	50%	30%	0.424
Age of onset			0.264
Mean ± SD	56.43 ± 9	61.07 ± 8.75	
(95% CI)	(48.11–64.75)	(56.22–65.91)	
Range:min-max	42.0–67.0	46.0–74.0	

SD: standard deviation; CI: confidence interval.

## Data Availability

Anonymized data not published within this article will be made available by request from any qualified investigator.
